# Exploitation of Modal Superposition Toward Forced Vibration Localization in a Coupled Symmetric Oscillator Array

**DOI:** 10.3390/s25103106

**Published:** 2025-05-14

**Authors:** Yannik Manz, Heiner Storck, André Gerlach, Norbert Hoffmann

**Affiliations:** 1 Hamburg University of Technology, Institute for Structural Dynamics, Schloßmühlendamm 30, 21073 Hamburg, Germany; norbert.hoffmann@tuhh.de; 2 Robert Bosch GmbH, Corporate Research (CR), Robert-Bosch-Campus 1, 71272 Renningen, Germany; heiner.storck@de.bosch.com (H.S.); andre.gerlach@de.bosch.com (A.G.); 3 Imperial College London, Mechanical Engineering, Exhibition Road, London SW7 2AZ, UK

**Keywords:** transducer arrays, coupled oscillators, forced vibration, localization, curve veering, modal superposition

## Abstract

In transducer arrays, symmetric grouping of identical elements is often employed to achieve uniform array performance. Such arrays can possess high coupling, preventing localized operation of individual transducers. This paper provides insight into how forced vibration localizes in a symmetric system of coupled oscillators. We use a simple lumped-parameter model of highly coupled oscillators derived from ultrasound transducer arrays. Forced vibration localization can be shown to be inversely related to the coupling strength between the oscillators. The results demonstrate how forced vibration in a coupled symmetric system may localize through modal superposition and how it may be tuned via the spacing of natural frequencies. Breaking the system’s symmetry leads to normal mode localization, which can be shown to affect the forced vibration response. The results reveal a variation in the system’s resonance frequency, attributed to curve veering effects.

## 1. Introduction

In array sensors, the localized vibration response of individual array transducers is a common goal with regard to array transmission performance. For uniform functionality, an array’s elements are often assumed to be equal and are arranged in a symmetric setup [1,2,3]. Since this is usually accompanied by tight geometric grouping, this setup imposes coupling between these elements. In vibration problems, it is well known that coupling leads to the distribution of a structure’s vibration properties over its domain. The modes become *extended*. In the special case of ordered systems that exhibit some sort of symmetry, this manifests in symmetric amplitudes of motion over the symmetry’s domain. Such extended modes make localized vibration of individual transducers difficult.

In a coupled system, free vibration localization may be observed once a system parameter is varied. Localization was first discussed in the field of solid-state physics [4,5] and has since been known as Anderson localization. Since then, similar phenomena have also been found to occur in structural dynamics problems. Localization in this context is characterized as the confinement of a mode’s amplitude to a local region of the structure. It is referred to as *normal mode localization*. Normal mode localization has been found to occur in cyclic systems once irregularities disturb their ordered states [6,7,8]. The phenomenon has been linked to curve veering, where the loci of two eigenvalues plotted over a parameter approach each other, then abruptly diverge without crossing [9]. Early reports on its occurrence were made in the context of plates and similar structures, where curve veering results in deformations of the nodal patterns [10,11,12,13]. The underlying concepts have since been investigated with regard to plates [14,15] and have been expanded to general cyclic groupings of oscillators [16,17]. More recently, attempts have been made to quantify the effects of curve veering, normal mode localization, and the mechanics of coupling [18,19,20,21,22,23]. Detailed reviews of localization phenomena in structural dynamics and their various implications for engineering structures can be found in [24,25].

The previously mentioned studies provide insight into free vibration of symmetric structures and the implications of disorder therein. Yet, few studies have connected the effects of normal mode localization and curve veering to a structure’s forced vibration response. Insights have been given based on a chain of coupled pendulums [26], but links between normal mode localization and forced vibration localization with regard to symmetric structures are otherwise scarce. Although some applications in micro-electromechanical system (MEMS) sensor arrays exploit localization effects introduced by disorder [27,28,29,30], these investigations are based on a removal of the underlying array’s symmetry. This allows the use of modal localization during operation [31,32]. Vibration localization has also been treated in ultrasound transducer arrays used for various sensing applications [33,34,35,36,37,38]. While a link between localization and the modal properties of an investigated array was provided in [39], such studies often focus on treating the forced response. Improvements to localization are typically proposed either by structural modifications or control algorithms [40,41,42,43,44]. Yet, skipping the treatment of free vibrations in these systems limits understanding, as extended modal properties due to an array’s symmetry and their relation to the observed forced response are not considered. Possible effects from normal mode localization cannot then be incorporated into the interpretation of results. In addition, specific array geometries are often used to provide insight into localization. This restricts the transferability of the presented results toward a more general treatment of forced vibration localization in coupled arrays.

The preceding studies deal with various variations of forced vibration localization in arrays. They either limit their focus to improving forced vibration localization in systems under excitation (skipping a relation to free vibration and the possible localization effects found therein) or deliberately remove the system’s symmetry to exploit modal localization effects during operation. We are left with the question of how modal properties contribute to the localization of forced vibration in coupled, symmetric systems and the possible influence of normal mode localization thereon. The treatment of this fundamental case is the scope of this paper.

For completeness, a brief reference to recent studies on localization and nonlinearity is in order. Cyclic groupings of nonlinear oscillators have been found to exhibit vibration localization even in perfectly symmetric systems without disorder [45,46,47,48]. This has been attributed to bifurcation phenomena arising from nonlinearity. While these investigations prove valuable, for example, in predicting the behavior of complex turbomachinery [49], transducer arrays are often operated under assumed linear conditions [1], and we focus our work on linear systems.

This paper uses a simple lumped-parameter model to investigate the forced vibration response of a highly coupled structure and its relation to modal properties. We set up a system of four active elements embedded on some arbitrary base. The system is chosen to be symmetric with regard to the active elements, and high coupling is introduced. The strength of coupling is used to vary the system’s setup. We excite the structure locally and examine its steady-state vibration response. Disorder is then introduced into the system, and its influence on the forced vibration response is investigated.

## 2. Methods and Model

### 2.1. Perturbation Theory

Mode localization is usually associated with two closely spaced eigenvalues of a structure’s eigenproblem. This is also the case in the system we are going to consider. Its analysis is commonly performed using perturbation methods. Perturbation theory with regard to structural dynamics problems is extensively covered in the literature, e.g., [18,50,51,52]. It allows the introduction of disorder into a system and the analysis of its effects on the system’s behavior. As perturbation theory is closely linked with the investigation of mode localization and we make use of its general concepts, we give a brief introduction to the topic here.

Consider a conservative mass-spring system. Its unperturbed eigenvalue problem, whose exact solutions are assumed to be known, is

(1) $$\left[K^{\left(0\right)}-\lambda^{\left(0\right)}M^{\left(0\right)}\right]{\hat{x}}^{\left(0\right)}=0,$$

where M and K denote the mass and stiffness matrix, respectively; $\hat{x}$ is a vector of system coordinates; and $\lambda ={\bar{\omega }}^{2}$ are the eigenvalues as squares of natural frequencies. The superscript ^(0)^ denotes the unperturbed state. We define a parameter ε that produces small corrections in K and M. These matrices become $K=K^{\left(0\right)}+\varepsilon V^{K}$ and $M=M^{\left(0\right)}+\varepsilon V^{M}$, where $V^{K}$ and $V^{M}$ are the perturbation operators of order 1. The perturbed eigenproblem then presents as

(2) $$\left[K-\lambda \;M\right]\hat{x}=\left[\left(K^{\left(0\right)}+\varepsilon V^{K}\right)-\lambda \left(M^{\left(0\right)}+\varepsilon V^{M}\right)\right]\hat{x}=0,$$

with perturbed solutions $\hat{x}$ and λ. Applying proportional perturbation, i.e., a perturbation operator V is chosen so that the system’s symmetry is retained, it can be shown that the system does not exhibit curve veering, and normal mode localization will not occur. The introduction of a reduced perturbation, breaking the symmetry of the system when evaluating its perturbed state, yields the conditions for mode localization and curve veering to occur. A similar result is achieved by removing the system’s initial symmetry. Disorder is introduced into the unperturbed state, and performing proportional perturbation then yields localization of the modes [9]. We use the latter case later on to investigate the implications of disorder in our model of a symmetric transducer array. For applications of the perturbation method to disordered cyclic systems, the reader is referred to [50,52,53,54]. A detailed discussion of general perturbation theory can be found in [55].

### 2.2. Frequency Response in Modal Context

Evaluation of the forced vibration response requires excitation of a structure. Taking the equation of motion of a mass-spring system, whose eigenproblem is given by Equation (1), we introduce an excitation vector F. In order to obtain finite amplitudes in resonance, damping is introduced into the system. Viscous damping may be chosen in stiffness-proportional form as C=βK, which retains modal diagonalization properties. We only consider steady-state vibration, for which the system’s equation of motion can be written as

(3) $$-\omega^{2}\;M\;\hat{x}+i\;\omega \;C\;\hat{x}+K\;\hat{x}=\hat{F},$$

where i and ω are an imaginary unit and the frequency of harmonic excitation, respectively, and amplitude values are denoted by a hat. For such cases, the frequency-response function (FRF) of the system maps the input (excitation) to the output (oscillator displacement). It is given as

(4) $$H\left(\omega \right)={\left(-\omega^{2}\;M+i\;\omega \;C+K\right)}^{-1}.$$

The general element in the FRF matrix, $H_{jl}\left(\omega \right)$, linking the input at oscillator *l* to the output at oscillator *j*, is

(5) $$H_{jl}\left(\omega \right)=\left(\frac{{\hat{x}}_{j}}{{\hat{F}}_{l}}\right),\;{\hat{F}}_{n}=0\;\;\mathrm{for}\;\;n\neq l.$$

To convert the problem to modal domain, we take a substitution $\hat{x}=\Phi \;\hat{q}$, where Φ is the modal matrix as a set of eigenvectors $\phi_{r}$. Modal transformation yields a *modal FRF*$H^{R}\left(\omega \right)$:

(6) $$H^{R}\left(\omega \right)={\left(-\omega^{2}\;M^{R}+i\;\omega \;C^{R}+K^{R}\right)}^{-1}.$$

The superscript ^R^ denotes the modal domain. $M^{R}$, $C^{R}$, and $K^{R}$ are the mass-normalized matrices of the form

(7) $$\begin{array}{c} \begin{array}{cc} M^{R} & =\Phi^{T}\;M\;\Phi ,\\ C^{R} & =\Phi^{T}\;C\;\Phi ,\\ K^{R} & =\Phi^{T}\;K\;\Phi , \end{array} \end{array}$$

with ^T^ denoting a transpose. Note that as $M^{R}$, $C^{R}$, and $K^{R}$ are diagonal matrices, $H^{R}\left(\omega \right)$ is a diagonal matrix as well. Modal displacements $\hat{q}$ are obtained by

(8) $$\hat{q}=H^{R}\left(\omega \right)\;{\hat{F}}^{R},$$

where ${\hat{F}}^{R}$ denotes an excitation vector in modal space given by

(9) $${\hat{F}}^{R}=\Phi^{T}\;\hat{F}.$$

Transferring these results back to the physical space, the *physical FRF* can be written as a superposition of its modal components:

(10) $$H\left(\omega \right)=\sum_{r=1}^{N}H_{r}\left(\omega \right),\;\;H_{r}\left(\omega \right)=\frac{\left(\phi_{r}⊗\phi_{r}\right)}{\left(-\omega^{2}\;M_{rr}^{R}+i\;\omega \;C_{rr}^{R}+K_{rr}^{R}\right)},$$

where $H_{r}\left(\omega \right)$ is the component of the *r*th mode and $\left(\;\cdot \;⊗\;\cdot \;\right)$ denotes an outer product. In discrete systems, as considered throughout this paper, the outer product is $u⊗v=u\;v^{T}$. $M_{jl}^{R}$, $C_{jl}^{R}$, and $K_{jl}^{R}$ correspond to the (j,l)th element in the mass-normalized matrices in modal space. Equation (10) is used in the next section to set up a modal contribution matrix.

### 2.3. Modal Contribution Matrix

Construction of the physical FRF from modal components allows the determination of modal contributions to coordinate displacements resulting from excitation. We use this concept during model analysis to evaluate modal participation in the localization of the system’s forced response. We take the contribution of the *r*th mode to the total displacement

(11) $${\hat{x}}_{r}=H_{r}\left(\omega \right)\;\hat{F}.$$

Normalization against the total physical displacement $\hat{x}$ leaves the relative contribution of the *r*th mode:

(12) $${\hat{x}}_{r}^{\mathrm{rel}}=\mathrm{diag}{\left(\hat{x}\right)}^{-1}\;{\hat{x}}_{r}.$$

We sum the magnitudes of the relative modal contributions per element *n*

(13) $${\hat{x}}_{n}^{\mathrm{rel}}=\sum_{r=1}^{N}\left|{\hat{x}}_{r}^{\mathrm{rel}}\left(n\right)\right|$$

and normalize the magnitude of the relative contribution of the *r*th mode, $\left|{\hat{x}}_{r}^{\mathrm{rel}}\right|$, against this sum. This obtains the relative contribution of mode *r* to the physical displacement at element *n*, normalized to range [0,1]:

(14) $${\hat{x}}_{r}^{\mathrm{rel},\mathrm{norm}}=\left[\begin{array}{c} {\hat{x}}_{r,1}^{\mathrm{rel},\mathrm{norm}}\\ \vdots \\ {\hat{x}}_{r,n}^{\mathrm{rel},\mathrm{norm}}\\ \vdots \\ {\hat{x}}_{r,N}^{\mathrm{rel},\mathrm{norm}} \end{array}\right],\;{\hat{x}}_{r,n}^{\mathrm{rel},\mathrm{norm}}=\frac{\left|{\hat{x}}_{r}^{\mathrm{rel}}\left(n\right)\right|}{\left|{\hat{x}}_{n}^{\mathrm{rel}}\right|},$$

where *N* is the number of degrees of freedom (DOFs). Putting this result into matrix notation yields a modal contribution matrix

(15) $$\mathrm{MCM}=\left[\begin{array}{ccccc} \; & \; & & \; & \\ {\hat{x}}_{1}^{\mathrm{rel},\mathrm{norm}} & \ldots & {\hat{x}}_{r}^{\mathrm{rel},\mathrm{norm}} & \ldots & {\hat{x}}_{N}^{\mathrm{rel},\mathrm{norm}}\\ \; & \; & & \; & \end{array}\right],$$

where each column gives the relative contributions of its mode to the oscillator displacements. From Equations (10) and (11), it can be seen that the contribution of each mode is weighted by the excitation, depending on the magnitude of its modal amplitude of motion at the excited DOF. Thus, the more a mode shape vector corresponds to the excitation vector, the higher its contribution to physical displacements. For steady-state vibration, the frequency of excitation determines the point of evaluation. This influences the relative modal contributions as well, which can be seen from the denominator in Equation (10). The closer an excitation frequency ω is to the damped natural frequency of a specific mode, the higher its relative contribution to the displacement. We use Equation (15) later on in a heatmap chart to visually quantify the modal contributions to the system’s forced response.

### 2.4. Lumped-Parameter Model: A Highly Coupled System

**Figure 1:**
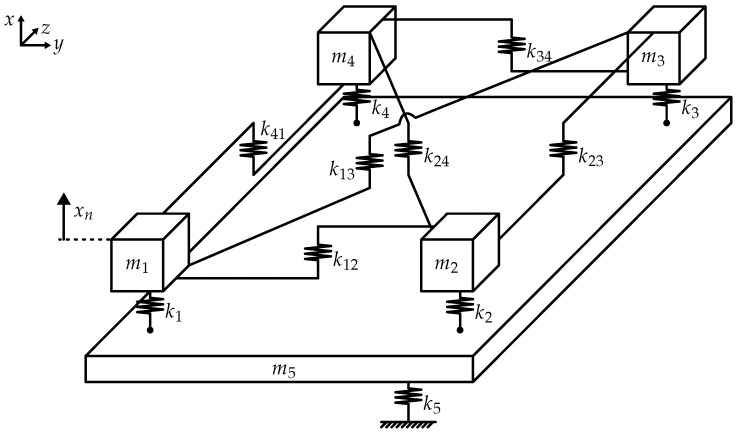
Lumped-parameter model: M-K system with 5 degrees of freedom (DOFs). Four oscillators (“elements”) are connected to a fifth oscillator (“base”). Oscillators 1–4 possess high inter-element coupling.

In this study, we use a lumped-parameter model to investigate forced vibration localization. Consider a simple mass-spring system with N=5 DOFs, as shown in Figure 1. It comprises four masses $m_{n}\;\left(n=1\ldots 4\right)$. They are connected to a 5th mass $m_{5}$ by springs $k_{n}$, forming four oscillators (or “elements”). Mass $m_{5}$ is referred to as “base” that is connected to ground by a spring $k_{5}$. The connection between the base and the oscillators induces base coupling among them. In addition, the four elements are coupled to each other by a total of six coupling springs $k_{nj}\;\left(n,j=1\ldots 4\right)$. The oscillators are considered *active*, with excitation possible for each element. Their relative displacements under forced vibration are used as a measurement of forced vibration localization. The base is introduced in the model to facilitate some arbitrary form of a *passive* structural component, which we cannot explicitly excite. Such a setup, where some active components are embedded in an arbitrary passive structure, is common in engineering applications. The system is chosen so that motion is possible only in the *x*-direction. Note the effect this has on the mounting of the coupling springs.

The system in Figure 1 is coupled, resulting in extended vibration properties. Its free vibration resembles that of a plate, which we demonstrate later. This is due to the system’s symmetry and its high degree of inter-element coupling.

To allow an investigation of the forced vibration response of this system, damping is introduced into the model. While the effects of damping on vibration localization were indicated in a study by Langley [56], we apply simple viscous damping. Determining its influence is not part of this work. We implement stiffness-proportional damping, as outlined in Section 2.2, of the form

(16) C=βK,

where β is a damping factor. Setting an excitation vector F, the equation of motion in matrix notation for the damped system is

(17) $$M\;\ddot{x}+C\;\dot{x}+K\;x=F.$$

Details are given in Appendix A.

#### 2.4.1. Symmetric Setup

As our investigation is motivated by symmetric transducer arrays, we define the conditions for a symmetric system with respect to oscillators 1–4. We choose the components as follows:

(18) $$\begin{array}{c} \begin{array}{cc} \; & m_{1}=m_{2}=m_{3}=m_{4}=m,\;\\ \; & m_{5}=a\;m,\\ \; & k_{1}=k_{2}=k_{3}=k_{4}=k,\;\\ \; & k_{5}=b\;k,\\ \; & k_{12}=k_{23}=k_{34}=k_{41}=k,\\ \; & k_{13}=k_{24}=0.9\;k. \end{array} \end{array}$$

Note that a factor of 0.9 is introduced in Equation (18) on the “diagonal” coupling springs to prevent a threefold degenerate eigenvalue. The parameters used throughout this study are listed in Table 1. All calculations were performed in *MATLAB (R2023b)* [57], unless stated otherwise.

In order to analyze the forced vibration localization of our model, a variation in our model is required. As is common in curve veering analyses, we choose to examine the eigenvalues as a function of a parameter. This parameter can, in general, be chosen at will. However, we require it to vary the system so that its symmetry is retained. We set the coupling springs $k_{nj}$ as variable and define their value with respect to a parameter ε as

(19) $$\begin{array}{c} \begin{array}{cc} \; & k_{12}=k_{23}=k_{34}=k_{41}=\varepsilon \;\cdot \;k\\ \; & k_{13}=k_{24}=\varepsilon \;\cdot \;0.9\;k. \end{array} \end{array}$$

The setup of Equation (19) maintains the system’s symmetry for all variations of ε. This method of variation is derived from perturbation methods, as described in Section 2.1. It is initially employed in the symmetric system.

#### 2.4.2. Disordered Model

In the second part of this paper, we investigate the effects of disorder on the forced vibration localization of our model. Two possible techniques for breaking the system’s symmetry were discussed in Section 2.1. We choose to apply disorder to the initial (and commonly assumed ordered) state of the system. The variation operator then needs to be chosen such that the system’s initial characteristics (i.e., those regarding symmetry in an ordered state) are preserved, allowing for a perturbation of the disordered state.

In Section 4, we apply this procedure to our 5-DOF model. We set $k_{1}=1.1\;k$ and retain the remaining definitions from Equation (18). This yields the disordered model conditions as follows:

(20) $$\begin{array}{c} \begin{array}{cc} \; & m_{1}=m_{2}=m_{3}=m_{4}=m,\;\\ \; & m_{5}=a\;m,\\ \; & k_{1}=1.1\;k,\\ \; & k_{2}=k_{3}=k_{4}=k,\;\\ \; & k_{5}=b\;k,\\ \; & k_{12}=k_{23}=k_{34}=k_{41}=k,\\ \; & k_{13}=k_{24}=0.9\;k. \end{array} \end{array}$$

Keeping Equation (19) for parameter variation allows for a symmetric perturbation of the disordered state.

## 3. Investigation of the Symmetric System: Results

This work seeks to understand localization properties of forced vibration in highly coupled array systems. We hope to uncover mechanisms that influence vibration localization. We start our investigation with the symmetric setup depicted in Figure 1 and described by Equation (18).

### 3.1. Parameter Variation: Increase in Coupling Stiffness

**Figure 2:**
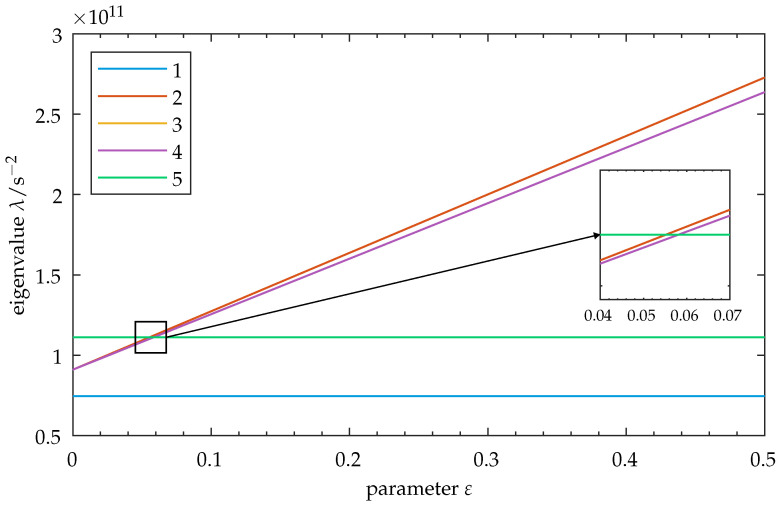
Symmetric model: Eigenvalues λ for modes 1–5 as a function of the parameter ε. Observe the increase in spacing between the eigenvalues as ε increases. The close-up shows crossing of the eigenvalue loci of modes 2, 3, and 4 through mode 5. As modes 3 and 4 form a degenerate mode pair, their eigenvalue loci overlap in this graphic. Note: As the factors a,b influence the vibration properties of mode 5, its eigenvalue may change by adapting these factors, thus moving the zone of eigenvalue crossing toward higher or lower values of ε. In this graphic, the upper limit for ε is set to 0.5 for clarity. Refer to Appendix B.1 for the full parameter range of this figure.

We take the undamped version of our model. Using Equations (18) and (19) in (17), we solve the characteristic eigenproblem as a function of the parameter ε. While these eigensolutions can be approximated with sufficient accuracy using the perturbation methods described in Section 2.1, our simple 5-DOF problem allows the computation of exact solutions without excessive numerical effort. Setting a range ε:[0,1.5], the eigenvalues λ as squares of angular natural frequencies $\bar{\omega }$ are shown as a function of the parameter ε in Figure 2. Note that the system exhibits base coupling for ε=0, as the elements are connected by the shared base $m_{5}$. We observe the crossing of mode 5 through the linearly increasing eigenvalue loci of modes 2, 3, and 4. Closer examination of the corresponding transition zone indeed shows an actual crossing of the involved modes—there is no curve veering present.

**Figure 3:**
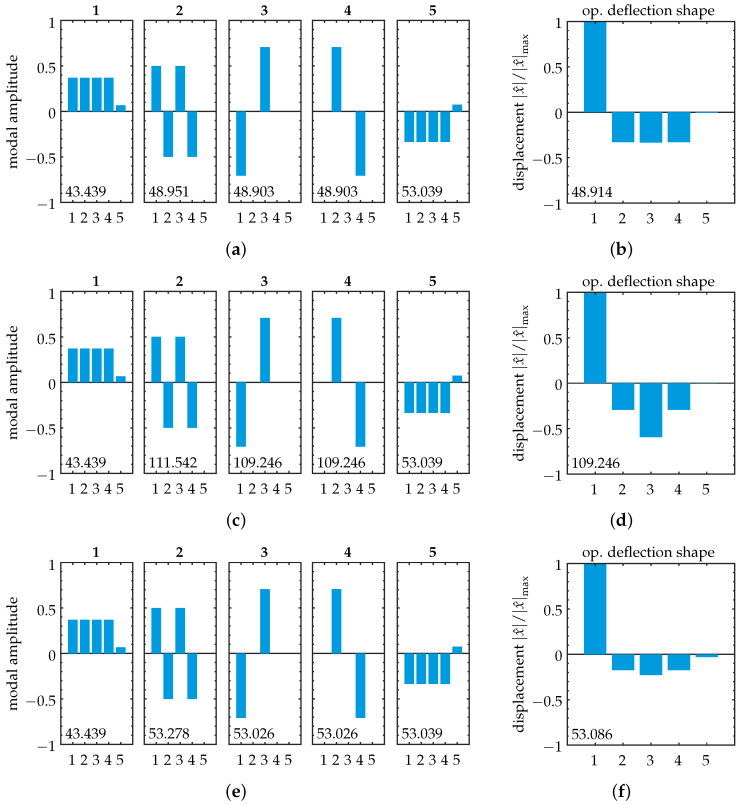
Symmetric model: Mass-normalized mode shapes (**left**) and simplified operational deflection shapes for excitation at oscillator 1 (**right**). The length of the vertical bars represents amplitudes of the corresponding oscillators. Natural frequencies in (**a**,**c**,**e**) and operating frequencies in (**b**,**d**,**f**) are given in [kHz] within each subplot. Various coupling cases are shown: (**a**,**b**) Weak coupling case, ε=0.01. (**c**,**d**) Strong coupling case, ε=1.1. (**e**,**f**) Case of eigenvalue crossing, ε=0.058. We observe unaltered mode shapes throughout parameter variation, as expected for a symmetric system. The operational deflection shapes do not resemble a specific mode shape; they are a result of modal superposition.

The mode shapes are examined next. Note that as we implement viscous damping, the damped system’s mode shapes correspond to those of the undamped system [59]. This feature allows us to perform simple computation of the undamped eigenproblem’s mode shapes and then use those for the interpretation of the damped forced vibration problem. The mode shapes corresponding to our model’s eigenvalues are shown in Figure 3 for three distinct values of ε. We observe no modal localization. The mode shapes present in extended form over the entire range of parameter variation. Note that the system’s symmetry is displayed in the motion amplitudes of the modes, as they exhibit symmetric behavior for oscillators 1–4. This resembles the nodal patterns of rectangular plate vibration, a feature of high inter-element coupling (compare with, e.g., [60] (p. 45)).

### 3.2. Effects of Coupling on the Forced Response

Next, we investigate the strength of coupling and examine its effects on the forced vibration response. We use the damped lumped-parameter model described in Section 2.4 and consider steady-state vibration for excitation at the first oscillator. Determining the FRF matrix and computing $\hat{x}=H\left(\omega \right)\;\hat{F}$ yields the forced response. It can be calculated for different values of ε. We treat three distinct cases.

#### 3.2.1. Case 1: Weak Coupling

**Figure 4:**
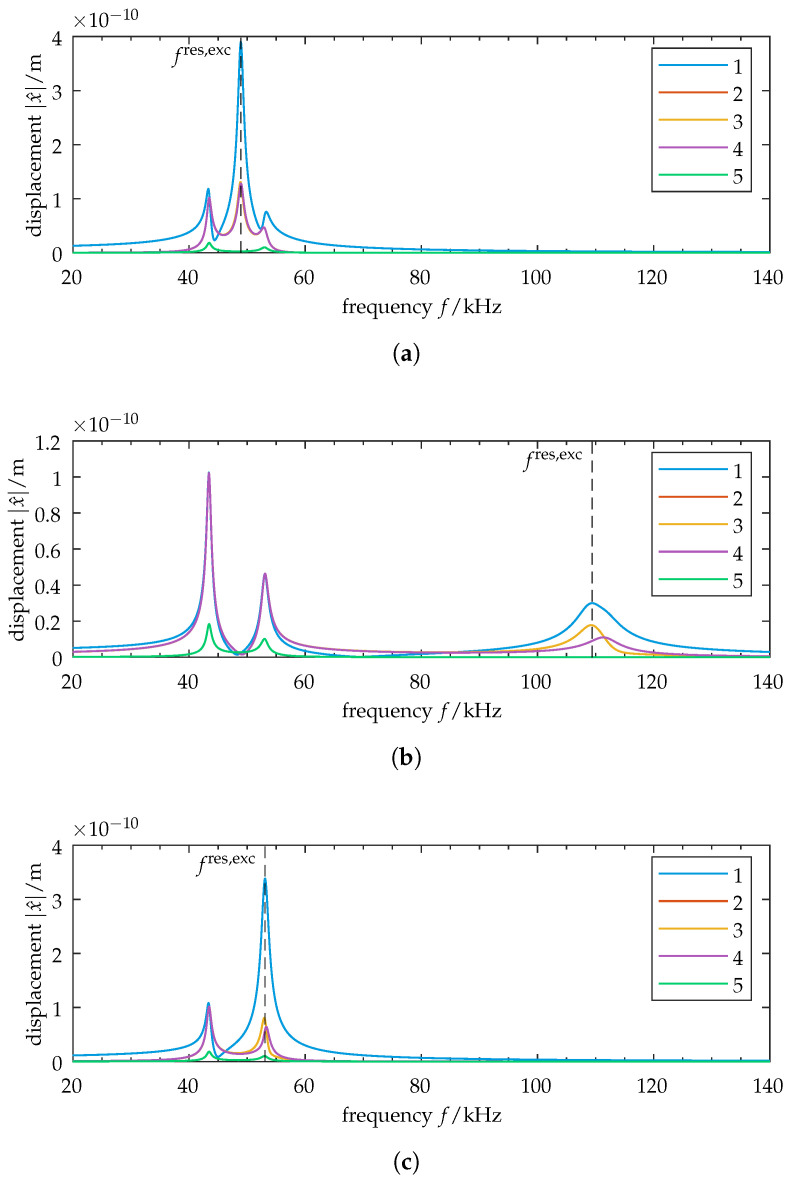
Symmetric model: Frequency responses of oscillators 1–5 for excitation at oscillator 1. The vertical dashed line indicates the resonance frequency of the excited oscillator $f^{\mathrm{res},\mathrm{exc}}$. Various coupling cases are shown: (**a**) Weak coupling case, ε=0.01. Relative displacement amplitudes at resonance frequency show vibration localization of the excited element. (**b**) Strong coupling case, ε=1.1. Evaluation is performed at the high-frequency resonance due to an increase in stiffness for strong inter-element coupling. The relative displacement amplitudes increase, reducing vibration localization compared to the weak coupling case. (**c**) Case of eigenvalue crossing, ε=0.058. Reduced relative displacement amplitudes show an increase in the vibration localization of the excited element. Note the scaling of the ordinates.

First, we take a look at the case of weak coupling, ε≪1. The frequency response is shown in Figure 4a. We evaluate vibration localization at resonance frequency of the excited oscillator, i.e., $\omega \;=\;\omega^{\mathrm{res},\mathrm{exc}}\;=\;2\pi f^{\mathrm{res},\mathrm{exc}}$. The corresponding complex amplitudes are shown in Table 2a. It can be seen that the vibration shows some degree of localization to the excited element, with the non-excited elements achieving amplitudes of ≈33%. Note that throughout this study, complex amplitude values are transformed into magnitude and phase angle. For our investigation of forced vibration localization, only the magnitudes are of interest. The phase angles are not evaluated. A visual interpretation of the resulting forced vibration localization is shown in Figure 3b, where the normalized displacement is plotted for each oscillator. By comparing it with the corresponding mode shape (cf. Figure 3a), it can be seen that the spatial distribution of the vibration amplitudes does not correspond to the distribution of one specific mode shape. We see the operational deflection shape as a result of modal superposition.

#### 3.2.2. Case 2: Strong Coupling

Next, we take a look at the case of strong coupling, ε≥1. The frequency response is shown in Figure 4b. Note that the point of resonance is defined here at the high-frequency peak due to an increase in stiffness for strong inter-element coupling. The vibration amplitudes at resonance are listed in Table 2b. We observe an increase in the maximum amplitude of the non-excited elements to ≈59%, reducing vibration localization. It should be noted that for oscillators 2 and 4, the vibration amplitude *decreases* compared to the weak coupling case. Again, the operational deflection shape in Figure 3d does not correspond to a specific mode shape of the structure.

#### 3.2.3. Case 3: Eigenvalue Crossing

In addition to the cases of weak and strong coupling, we choose our parameter so that it coincides with a point of eigenvalue crossing at ε=0.058 (cf. Figure 2, close-up). The frequency response for this setup is shown in Figure 4c. The corresponding vibration amplitudes are shown in Table 2c and visualized in Figure 3f. Increased vibration localization to the excited element can be observed, with a maximum amplitude at the non-excited elements of ≈23%.

The three cases studied above show that the forced vibration localization of the system depends on the strength of coupling between its elements. The relationship may be quantified by plotting the relative displacement amplitudes (as a measure of vibration localization) as a function of the parameter ε, representing the coupling strength. The corresponding figure is shown in Appendix C. In general, an increase in the coupling strength reduces vibration localization. This is particularly pronounced for weak coupling (i.e., small values of ε). In addition, it is possible to determine a coupling strength where the eigenvalues of the system intersect, resulting in increased localization of the vibration amplitudes to the excited element.

### 3.3. Relationship of Forced Vibration Localization to Modal Composition

**Figure 5:**
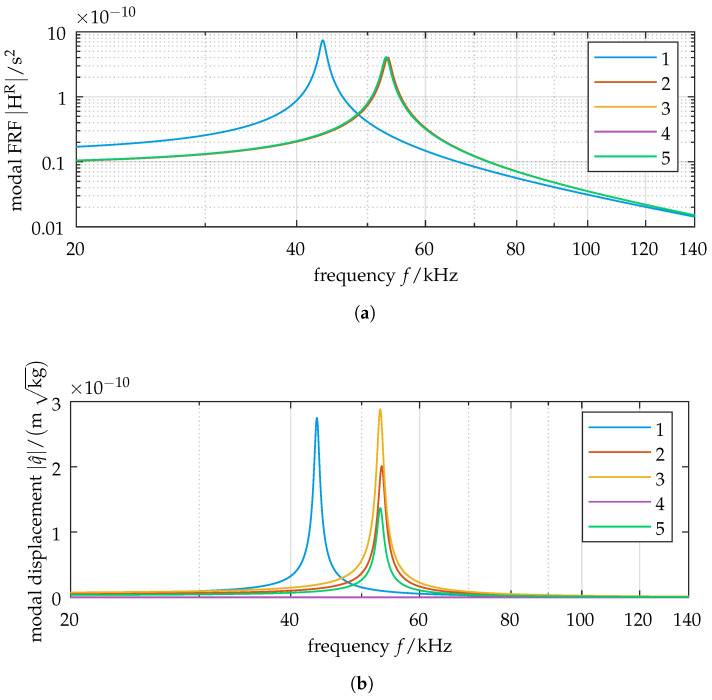
Modal treatment of the symmetric model: Case of eigenvalue crossing at ε=0.058. (**a**) Modal frequency-response function (FRF) for modes 1–5. We see almost equal damped natural frequencies for modes 2 through 5. (**b**) Modal displacement amplitudes for modes 1–5. The relative modal displacements are scaled due to modal weighting by the excitation vector. The damped natural frequencies remain unchanged. We observe zero displacement for mode 4 as a result of orthogonality between mode 4’s eigenvector and the excitation vector. (Note the different scales of the ordinates: (**a**) logarithmic, (**b**) linear. Grid lines are shown for log-scaled axes.)

The composition of the vibration response is analyzed next, using case 3 (eigenvalue crossing). Here, the natural frequencies of modes 2 through 5 show close proximity. The excitation frequency at resonance is thus very close to the damped natural frequencies of those modes. From the composition of the FRF as a superposition of its involved modes (cf. Equation (10)), we expect this to lead to a similar weighting of the modal contributions. The modal frequency response is shown in Figure 5a. We observe almost equal modal amplitudes for modes 2 through 5 and the corresponding close frequencies of those modes. To quantify the influence of excitation on modal participation, the modal displacement is calculated using Equation (8), and the corresponding frequency response is shown in Figure 5b. We observe some key changes compared to the modal FRF. First, and most notably, mode 4 disappears (${\hat{q}}_{4}\left(\omega \right)=0$). Mode 4’s mode shape vector (cf. Figure 3e) is orthogonal to the excitation vector. The contribution of mode 4 to displacement is thus weighted to zero. Second, we see a notable difference in the amplitudes of the modes with close frequencies due to the scaling of the mode shape vectors. The closer a mode shape vector corresponds to the excitation vector, the higher it is weighted in the modal force representation by Equation (9).

**Figure 6:**
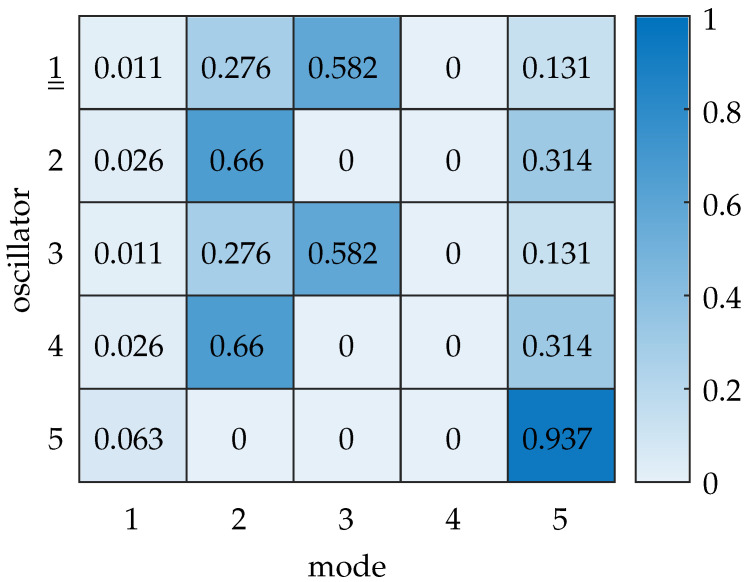
Heatmap of the modal contribution matrix for ε=0.058, with excitation at oscillator 1. The color bar indicates the normalized contribution of each mode ${\hat{x}}_{r,n}^{\mathrm{rel},\mathrm{norm}}$ to the displacement per oscillator. The double underline indicates the excited oscillator. Mode 3 contributes the most to the displacement at oscillator 1, followed by mode 2 and mode 5. The respective contribution ratios are related to the modal displacement amplitudes shown in Figure 5b.

The previously determined differences in the modal displacements are related to the physical displacements of the oscillators by modal substitution $\hat{x}=\Phi \;\hat{q}$. We quantify this relationship in detail via the modal contribution matrix. The heatmap chart for an excitation frequency $\omega \;=\;\omega^{\mathrm{res},\mathrm{exc}}$ is shown in Figure 6. We observe that for the excited oscillator 1, mode 3 contributes more than 50% to the element’s displacement. Mode 2 contributes ≈ 27%, and mode 5 contributes ≈ 13%. As the natural frequencies of these modes are closely spaced in this case, the modal contributions are dominated by the relative amplitudes of motion of the mode shapes (as seen in Figure 5b).

**A note on damping**
  Previously, it was determined that modal contributions are influenced by two factors: modal weighting due to the agreement between the mode shape vector and the excitation vector, and modal weighting due to the agreement between a mode’s damped natural frequency and the excitation frequency. Increasing modal damping yields well-known effects: the damped natural frequency of the system is lowered, and the displacement amplitude is reduced, increasing displacement bandwidth. In the context of modal superposition, these effects may cause a more equal contribution of modes with close (but not necessarily equal) frequencies. In other words, as damping increases, a positive effect on vibration localization is expected. This can be demonstrated by increasing the model damping (cf. Table 1), e.g., by a factor of 10. Considering the case of eigenvalue crossing (ε=0.058), a maximum amplitude at the non-excited elements of ≈15% is observed, increasing vibration localization compared to the lower-damped case treated in Section 3.2.3 (cf. Table 2c, where the maximum amplitude at the non-excited elements is ≈23%).

### 3.4. Interchange of Excitation—Retained Symmetry

As our system is symmetric, the interchange of excitation (e.g., from input at oscillator 1 to oscillator 2) retains the vibration localization properties on a relative scale between the elements. However, we need to keep in mind, in this context, that the *imprint* of the forced vibration response remains: the relative amplitude value at an observed oscillator may vary depending on the excited element. This can be imagined as a rotation of the entire system’s vibration state over the system’s central axis of symmetry. Refer to Appendix D for a visualization.

### 3.5. Complete Vibration Localization in a Symmetric System

The previous sections dealt with the vibration response resulting from local excitation. It was shown that modal superposition leads to confined vibration, even though the coupled system does not have any mode corresponding to localized vibration at the excitation point. We are left with the question of whether it is possible to completely confine the vibration to a single oscillator. This analysis requires us to address the *inverse* problem. We find the inverse FRF, which we call Y(ω):

(21) $$Y\left(\omega \right)={\left[H(\omega )\right]}^{-1}\equiv \left(-\omega^{2}\;M+i\;\omega \;C+K\right).$$

By setting a displacement vector of ${\hat{x}}_{\mathrm{target}}={\left[\begin{array}{ccccc} 1 & 0 & 0 & 0 & 0 \end{array}\right]}^{T}$ and using $\hat{F}=Y\left(\omega \right)\;{\hat{x}}_{\mathrm{target}}$, the required excitation vector can be retrieved. It should be noted that for our 5-DOF system, $\hat{F}$ contains complex values. The normalized result is presented in Table 3. Note the requirement of excitation on *all* DOFs. In the setup of our model, we defined the base $m_{5}$ as a *passive* component where explicit excitation is not possible. This requires the excitation vector to be zero for the fifth oscillator, $\hat{F}\left(5\right)\overset{!}{=}0$. The composition of the system’s matrices (cf. Appendix A) shows that this condition can only be met if $K_{51}=\left(-k_{1}\right)=0$. Since the system’s setup does not allow this without significantly influencing its characteristics, there is no complete vibration localization in the symmetric system.

Further insight into complete vibration localization is gained by considering only the relative displacements of the active elements 1–4. This approach may be beneficial if we are not interested in the relative displacement of the passive base and do not need to enforce it to zero as above. A reduced target displacement vector ${\hat{x}}_{\mathrm{target}}^{\mathrm{red}}={\left[\begin{array}{cccc} 1 & 0 & 0 & 0 \end{array}\right]}^{T}$ is applied, where the displacement of oscillator 5 is omitted. In order to obtain the required excitation vector, a reduced FRF and its inverse are necessary. In general, a reduction of the system’s FRF is performed by removing its $\bar{l}$th row and column, corresponding to the omitted DOF. The reduced FRF is written as

(22) $$H^{\mathrm{red}}\left(\omega \right)=H_{\bar{l}\;\bar{l}}\left(\omega \right).$$

For our model, we remove the row and column associated with the base $m_{5}$ and set $\bar{l}=5$. Computation of the inverse reduced FRF then yields

(23) $$Y^{\mathrm{red}}\left(\omega \right)={\left[H^{\mathrm{red}}\left(\omega \right)\right]}^{-1}.$$

Using ${\hat{F}}^{\mathrm{red}}=Y^{\mathrm{red}}\left(\omega \right)\;{\hat{x}}_{\mathrm{target}}^{\mathrm{red}}$ yields the required excitation vector for complete vibration localization within the active elements. Expanding this vector and including zero excitation at the base allows the computation of the entire system’s displacements. The normalized results are presented in Table 4. The left side shows the complex amplitudes for the required reduced excitation vector. On the right side, this vector is expanded by including zero excitation at the base. It is then used to calculate the displacements for the entire system. It can be seen that the displacement amplitudes for the active elements 1–4 are completely localized without excitation of the base. However, this leaves the displacement of the fifth oscillator as expected.

A note on robustness: As the proposed approaches for complete vibration localization require the computation of an inverse FRF, their sensitivity to parameter uncertainties needs to be kept in mind. Small deviations of the implemented model from the actual system may result in a mismatch between the assumed FRF and its actual counterpart. This, in turn, may influence the results obtained when extracting a target excitation vector and applying it to complete forced vibration localization.

## 4. Breaking System Symmetry: Introducing Disorder

In the next step, we break the symmetry of our system. It is well known from theory that the introduction of disorder into an otherwise symmetric structural dynamics problem can cause normal mode localization, where vibration becomes confined to an enclosed region. This phenomenon might be useful for localizing the forced vibration response in our model of a transducer array. Therefore, the effects of disorder are investigated.

### 4.1. Modal Properties

**Figure 7:**
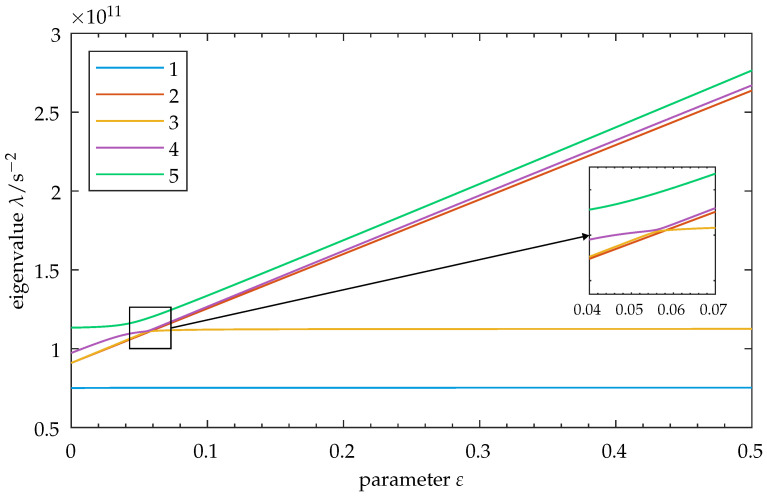
Disordered model: Eigenvalues λ for modes 1–5 as a function of parameter ε. Disorder is introduced into the initial state (cf. Equation (20)). Observe the increase in the spacing of the eigenvalues and their exchange of relative positions as ε increases. The close-up shows the transition zone, where curve veering can be observed for mode 5 and between modes 4 and 3. Curve crossing is then seen for modes 3 through 2. The degenerate mode pair from the symmetric case (cf. Figure 2) is not present here, as the degeneracy is resolved by introducing disorder. Note: As the factors a,b influence the vibration properties of mode 5, its eigenvalue may change by adapting these factors, thus moving the transition zone toward higher or lower values of ε. In this graphic, the upper limit for ε is set to 0.5 for clarity. Refer to Appendix B.2 for the full parameter range of this figure.

We introduce disorder on oscillator 1, as described in Section 2.4, Equation (20). The system is perturbed, and its eigenvalues are calculated as a function of the parameter. The resulting graph is shown in Figure 7. Some important features can be observed. At ε≈0.04, curve veering can be observed between modes 4 and 5. This is consistent with the introduction of disorder. Furthermore, there is modal interaction at ε≈0.055. A swap of trajectories for modes 3 and 4 can be seen, preventing a crossing at first. Upon detailed inspection of the transition zone, it can be seen that a crossing of modes 3 through 2 occurs after the trajectories of modes 3 and 4 are swapped. This differs from the symmetric case in Figure 2, where only eigenvalue crossings occurred.

**Figure 8:**
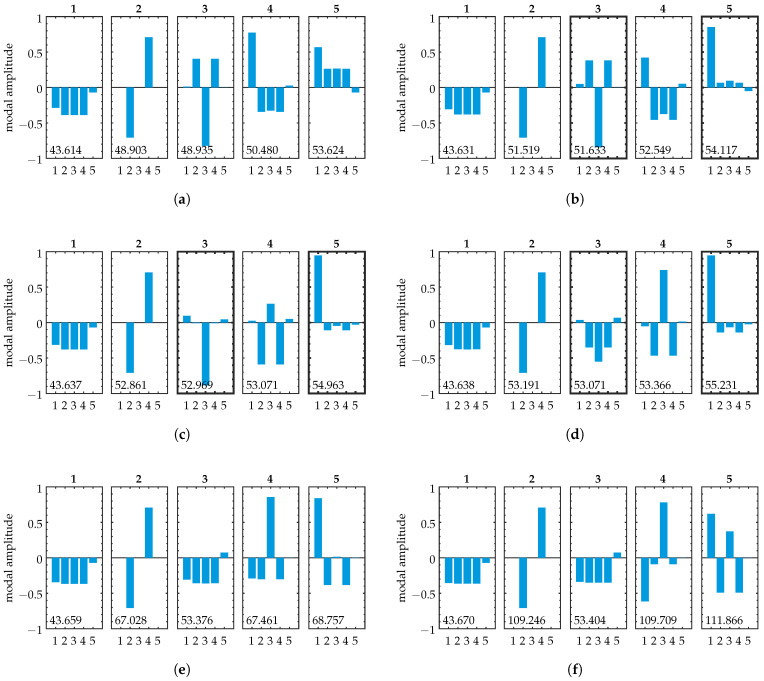
Disordered model: Mode shapes for various values of ε. Disorder is introduced into the initial state at oscillator 1 (cf. Equation (20)). The length of the vertical bars represents amplitudes of the corresponding oscillators in modal domain. Natural frequencies are given in [kHz] within each subplot. (**a**) ε=0.01, (**b**) ε=0.04, (**c**) ε=0.056, (**d**) ε=0.6, (**e**) ε=0.25, (**f**) ε=1.1. (**b**–**d**) show the transition zone, where curve veering and normal mode localization occur (cf. Figure 7, close-up). Compare (**a**,**e**) to see the swap of mode shapes 3, 4, and 5 with modes 4, 5, and 3, respectively (note that the shapes show increasing variation between (**a**) and (**e**,**f**), as their composition is influenced by the increase in ε). This is accompanied by normal mode localization in the transition zone for mode 5 (localization to oscillator 1) and mode 3 (localization to oscillator 3), as indicated by black boxes in (**b**–**d**).

The mode shapes throughout the transition zone are shown in Figure 8. We can observe the exchange of the properties of several modes. By comparing Figure 8a,e, we can see that the initial shape of mode 5 is transferred to mode 3. The initial shapes of modes 3 and 4 are transferred to modes 4 and 5, respectively (note that the shapes show slight variation between Figure 8a and Figure 8e,f, as their composition is influenced by the increase in ε). The transfer of modal properties occurs throughout the transition zone and is accompanied by localization behavior. Mode 5 shows strong localization to oscillator 1, and mode 3 displays localization to oscillator 3. We compare the weakly coupled case in Figure 8a to the symmetric equivalent in Figure 3a. In the disordered case, the vibration properties are changed for mode shapes with participation of the disordered oscillator 1. Mode shapes without participation of the disordered oscillator remain unchanged.

### 4.2. Forced Vibration in the Transition Zone

**Figure 9:**
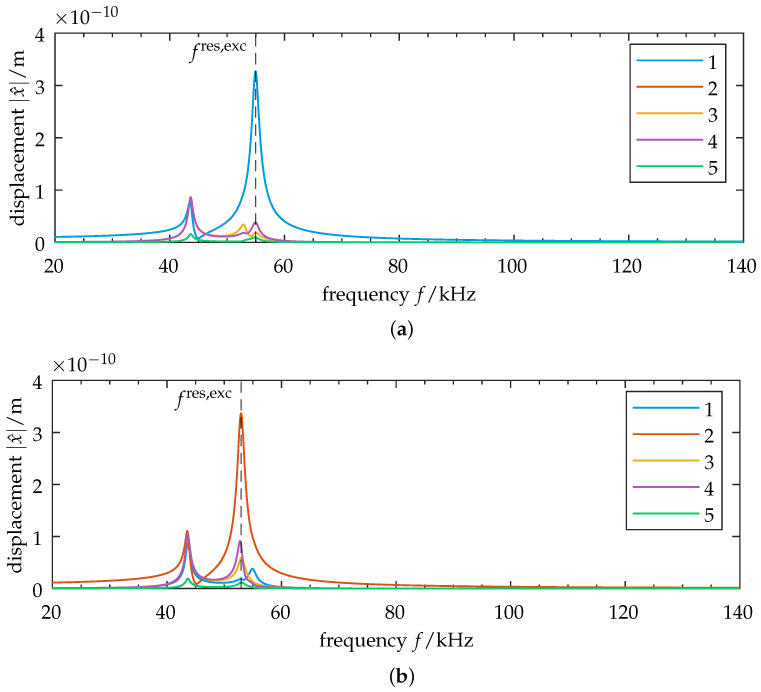
Disordered model: Frequency responses of oscillators 1–5 for ε=0.056, and different points of excitation. Disorder is introduced into the initial state (cf. Equation (20)). The vertical dashed line marks the resonance frequency of the excited oscillator $f^{\mathrm{res},\mathrm{exc}}$. (**a**) Excitation at oscillator 1, which shows significant localization. (**b**) Excitation at oscillator 2. Observe the change in the relative amplitudes of the non-excited elements at resonance frequency. (**b**) shows significantly higher relative displacements at excitation frequency and thus reduced vibration localization when compared with (**a**).

We are now interested in the forced vibration localization of the disordered model. As we have already determined, forced vibration localization is particularly advantageous for closely spaced eigenvalues, so we investigate the transition zone. The frequency response for ε=0.056 is shown in Figure 9a. The normalized displacement values at $\omega \;=\;\omega^{\mathrm{res},\mathrm{exc}}$ are given in Table 5 and visualized in Appendix E. Strong vibration localization can be confirmed. The displacement amplitudes at the non-excited elements drop to ≈12% (compared to ≈23% for the symmetric case at eigenvalue crossing). This shows that introducing disorder changes the forced vibration response. We see an increased vibration localization under excitation. Since our system now shows normal mode localization and curve veering, the modal composition of the forced response is of special interest in determining its effects thereon.

### 4.3. Modal Composition in the Disordered Model

**Figure 10:**
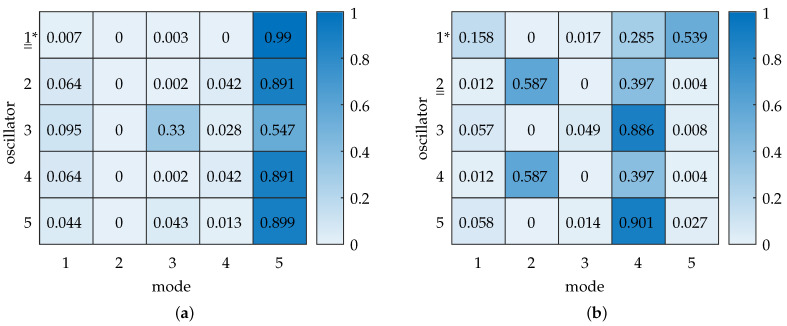
Disordered model: Heatmaps of modal contribution matrices for ε=0.056. The color bar indicates the normalized contribution of each mode ${\hat{x}}_{r,n}^{\mathrm{rel},\mathrm{norm}}$ to the displacement per oscillator. (*) denotes the disordered oscillator, and a double underline indicates the excited oscillator. (**a**) Excitation at oscillator 1. Mode 5 shows an almost exclusive contribution to the displacement at the excited oscillator. We note the distribution at oscillator 3, where modes 5 and 3 show significant contributions. This is caused by localization effects on oscillator 3 in mode 3. (**b**) Excitation at oscillator 2. Mode 2 shows the highest contribution to the displacement at oscillator 2. Note the difference in relative modal contributions when comparing this heatmap with the excitation at oscillator 1 (**a**). In (**b**), modes 2, 4, and 5 now each show significant modal contributions to displacements in at least one oscillator.

The modal composition of the forced vibration response in the transition zone is shown in Figure 10a. Mode 5 contributes around 99% to the displacement at oscillator 1. Considering strong normal mode localization of this mode to oscillator 1, its excitation leads to strong agreement between the *localized* mode shape vector and the excitation vector. This, in turn, explains the strong contributions of mode 5 to the displacements.

In addition, we observe a special modal composition at oscillator 3 that stands out, as there is no bulk of contribution by mode 5. Instead, mode 3 is also significantly involved in contributing to the displacement of oscillator 3. Recalling localization in mode 3 to oscillator 3 (cf. Figure 8c), the participation of mode 3 in the displacement of oscillator 3 can be explained.

The modal contribution matrix confirms that, as normal mode localization occurs in the disordered system, the excitation of a localized DOF leads to a domination of the forced vibration by the corresponding localized mode. However, for different oscillators, we can also see increased contributions of other modes. This leaves the question of how an exchange of excitation influences vibration.

### 4.4. Exchange of Excitation

As our initial system is now disordered, we expect a variation in the system’s behavior upon interchanging excitation throughout the four active oscillators. We examine the system for a switch of excitation to oscillator 2, whose DOF shows little localization in the transition zone during parameter variation. Applying the corresponding excitation vector yields the frequency response shown in Figure 9b. It shows reduced vibration localization at $\omega \;=\;\omega^{\mathrm{res},\mathrm{exc}}$. The values are listed in Table 5 and visualized in Appendix E. Comparing this to the symmetric case (cf. Table 2c), a difference of ≈1% shows that the vibration localization is only slightly less pronounced for the disordered system. By looking at the modal contribution matrix shown in Figure 10b, we see that the disordered system’s forced vibration localization is composed mainly of modes 2 and 4 for the excitation at oscillator 2. This shows significantly different modal contributions compared to the disordered system’s excitation at oscillator 1.

Further variation of the excitation vector shows that for excitation at oscillator 3, an increased vibration localization can be observed. This is attributed to the mode shape localization to its DOF, as is the case for the excitation at oscillator 1. The normalized displacement amplitudes for the individual excitations at elements 1–4 are given in Table 5. Their operational deflection shapes are shown in Appendix E.

The results show that in a disordered system, an exchange of excitation causes the forced vibration localization to vary. Strong agreement between the excitation vector and the localized normal mode in the transition zone causes the forced response to strongly localize. Excitation at oscillators that are not strongly influenced by normal mode localization shows only a small influence on the localization of the forced vibration response.

**On resonance frequency**
  A remark should be made regarding resonance frequency. As we focused our research on the system’s behavior *at resonance frequency*, the frequency itself was not considered. Table 5 shows the values for resonance frequency $f^{\mathrm{res},\mathrm{exc}}$ as the excitation of the disordered model is exchanged. We can observe a change in resonance frequency that is most significant for the disordered oscillator. This can be attributed to curve veering, where, by definition, the eigenvalues of two modes cannot coincide during interaction. As the excitation of different oscillators mainly involves the corresponding localized normal modes, the resonance frequency changes accordingly. This frequency deviation relates to the amount of localization and curve veering (and, as a consequence, to the chosen parameter point ε).

## 5. Discussion

This paper uses a simple lumped-parameter model of a highly coupled, symmetric oscillator array to study the localization of its forced vibration response. The model’s symmetry is then broken by introducing disorder, and the resulting phenomena of normal mode localization and curve veering are investigated regarding their effects on forced vibration localization.

The results provide insight into general forced vibration localization in a coupled, symmetric system. Our study shows that with an increase in the system’s inter-element coupling strength, an inverse relation to the degree of localization in the forced response is observed. The symmetric setup of the system allows the determination of a point where the system’s eigenvalues are closely spaced. Here, its forced response shows increased vibration localization, an effect attributed to the advantageous superposition of the system’s symmetric modes. Manipulation of the coupling strength between elements thus provides a possibility to influence the system’s forced vibration response. This result is important to note when working toward individually driven transducers in highly coupled sensor arrays. It may be used to decrease mechanical crosstalk between active and passive transducer elements. Additional emphasis should be placed on our results regarding complete vibration localization. We showed that by using an inverse frequency-response function, it is possible to determine an excitation vector that completely localizes the forced vibration to the excited oscillator (cf. Section 3.5). This is accompanied by a necessity to excite *all* available degrees of freedom. Our system’s setup included a passive structural component without the possibility for excitation—a common case in many engineering structures. For such structures, our results imply that complete vibration localization is not possible, limiting the achievable amount of forced vibration localization. This may affect the possible reduction in mechanical crosstalk in array systems. By applying a reduced FRF and considering vibration localization only within the active oscillators of our system, we showed that it becomes possible to fully localize the forced vibration to an active element without excitation of the passive structure. This is, however, accompanied by a relative displacement of the passive component. Such an approach allows control of the vibration localization properties in symmetric array systems if the relative displacement of a passive component does not disturb the intended functionality and can be omitted. It may be applied to increase the reduction in mechanical crosstalk in the array.

Further insight was gained from breaking the system’s symmetry and investigating the resulting effects on forced vibration localization. As expected from structural irregularities within symmetric systems, we observed curve veering of the system’s eigenloci. It occurred in a transition zone where at least two eigenvalues became closely spaced. The exchange of modal properties within that zone led to normal mode localization. We demonstrated the effects of normal mode localization on the forced response. Our results showed a dependence on the excited oscillator: By exciting an oscillator that exhibited localization within the normal modes, it was shown that the forced vibration response increased its localization, as the localized normal mode showed significant contribution. In contrast, excitation of non-localized oscillators showed little effect on the amount of forced vibration localization compared with the symmetric system. In addition, we demonstrated the effects of normal mode localization on the resonance frequency of the excited oscillator. A change in resonance frequency related to the excited oscillator was observed as a consequence of curve veering. This result is not intuitive and is particularly important in systems where the excitation frequency is small-banded (or mono-frequency is employed). In such cases, a variation in the resonance frequency could lead to a mismatch between the excitation frequency and the resonance frequency of the structure. This may reduce performance for transducer arrays where operation at resonance is required, e.g., to achieve increased displacement amplitudes during operation.

Contrary to initial intuition when dealing with normal mode localization, its effects are only advantageous regarding forced vibration localization if the focus is on a single transducer and its forced vibration localization. Requirements to interchange excitation—a common case in array systems where uniform transmission characteristics are targeted—prevent the exploitation of normal mode localization, as the system’s vibration properties may vary strongly depending on the excited oscillator.

We expect the results of this study to be valuable for the design of symmetric sensor arrays. In this context, emphasis should be placed on the implications of our results for system optimization. At eigenvalue proximity, the symmetric system was shown to have increased forced vibration localization. We showed, in turn, that this area was particularly affected by structural irregularity, becoming a transition zone in the disordered model. For an assumed symmetric system with optimization targeted toward increased forced vibration localization (e.g., to reduce mechanical crosstalk), a point of close eigenvalue proximity would be chosen in the design phase. Structural irregularity would then exert a particularly strong influence due to curve veering and normal mode localization phenomena associated with eigenvalue proximity in the transition zone. The system’s optimization in a symmetric state thus requires precise control over its symmetry conditions. A tolerance-based approach would thus be deemed necessary to account for possible deviations.

To gain further insight into vibration localization behavior, the investigations conducted in this study may be expanded. We plan to transfer the problem to time domain to study the effect of modal interaction on transient behavior in highly coupled systems. We hope to gain insight into array system dynamics where excitation is time-dependent. Furthermore, a separate treatment of the influence of damping on forced vibration localization may reveal other paths of controlling localization in coupled arrays.

In addition to the use of our results for transducer array design optimization, the results regarding disorder could be used to detect structural irregularities within existing sensor arrays. The relative increase in vibration localization when exciting a disordered degree of freedom, accompanied by a change in the expected resonance frequency, may be exploited for this.

## 6. Conclusions

This paper is motivated by transducer arrays, where striving for equal transducer performance often requires a symmetric setup. Such arrays may possess coupling between their elements, making localized operation of individual transducer elements difficult. We used a coupled lumped-parameter model to investigate localization of the forced response in this context. The results for the symmetric case showed that forced vibration localization is possible in symmetric systems, even though their modes are extended over the entire structure. Although the strength of coupling is, in general, inversely related to the achievable amount of vibration localization, we demonstrated that it is possible to choose a coupling parameter such that the system’s eigenvalues become close. This leads to an advantageous superposition of the system’s modes that can be exploited for forced vibration localization of the system. We also showed that the use of an inverse frequency-response function enables full confinement of the vibration to an individual oscillator by extracting a suitable excitation vector. However, this requires access to the excitation at all degrees of freedom of the structure, something we explicitly denied in the setup of our model, as will be common in many engineering structures. This limitation can be overcome by applying a reduced frequency-response function if the relative displacement of the passive structural component is not of interest. Complete vibration localization to an active oscillator is then possible.

The introduction of disorder into the model showed normal mode localization and curve veering. We demonstrated how the forced vibration response is influenced by normal mode localization and how this translates to a dependence of the forced vibration on the point of excitation. Increased vibration localization was observed for excitation at an oscillator that showed modal localization. For excitation at non-localized oscillators, we observed little effect on forced response localization. We further showed that breaking the system’s symmetry leads to a dependence of the system’s resonance frequency on the location of excitation. This comes as a consequence of curve veering phenomena that accompany normal mode localization in disordered systems. It is of particular importance in systems where small-band excitation requires close control of the system’s behavior under resonance.

## Figures and Tables

**Table 1:** Model parameters. Overview of the model parameters used throughout this study, unless specified otherwise. The target frequency is chosen to represent air-coupled ultrasound transducers, where operating frequencies of 40–60 kHz are common [58] (p. 247). All other parameters are chosen arbitrarily. Note: The implemented damping can be considered weak.

Measure	Symbol	Unit	Value
Target frequency	$f_{0}$	Hz	48,000
Mass	*m*	kg	1
Stiffness	*k*	N m^−1^	${\left(2\pi f_{0}\right)}^{2}\;m$
Damping (prop. stiffness)	β	s	$0.02/\left(2\pi f_{0}\right)$
Excitation amplitude	$\hat{F}$	N	1
Factor mass (base)	*a*	-	100
Factor stiffness (base)	*b*	-	100

**Table 2:** Complex displacement amplitudes for $\omega \;=\;\omega^{\mathrm{res},\mathrm{exc}}$. Three cases for the coupling parameter ε. (**a**) Case 1, weak coupling: The vibration amplitudes of the non-excited elements (oscillators 2–4) reach ≈33% of the amplitude of the excited element. (**b**) Case 2, strong coupling: The vibration amplitudes of the non-excited elements reach up to ≈59% of the amplitude of the excited element. Reduced localization compared to the weak coupling case. (**c**) Case 3, crossing: The vibration amplitudes of the non-excited elements reach ≈23% of the amplitude of the excited element. This shows a significant increase in localization compared to the weak and strong coupling cases. Note: The phase angles are displayed for completeness; they are not evaluated in this study.

		(a) ε=0.01		(b) ε=1.1		(c) ε=0.058	
Oscillator	$\hat{F}$	$\left|\hat{x}\right|/{\left|\hat{x}\right|}_{\max}$	$\psi_{\hat{x}}{/}^{\circ }$	$\left|\hat{x}\right|/{\left|\hat{x}\right|}_{\max}$	$\psi_{\hat{x}}{/}^{\circ }$	$\left|\hat{x}\right|/{\left|\hat{x}\right|}_{\max}$	$\psi_{\hat{x}}{/}^{\circ }$
1	1	1	−89.4	1	−80.6	1	−89.7
2	0	0.328	94.3	0.293	135.1	0.173	129.1
3	0	0.334	83.5	0.594	74.0	0.229	56.5
4	0	0.328	94.3	0.293	135.1	0.293	135.1
5	0	0.007	177.8	0.000	3.9	0.030	89.3

**Table 3:** Complex excitation amplitudes for $\omega \;=\;\omega^{\mathrm{res},\mathrm{exc}}$ and complete localization to oscillator 1: ${\hat{x}}_{\mathrm{target}}={\left[\begin{array}{ccccc} 1 & 0 & 0 & 0 & 0 \end{array}\right]}^{T}$. Case of close frequencies at crossing, ε=0.058. We see a requirement to excite the base (oscillator 5) in order to achieve complete vibration localization to a single oscillator.

		Required Excitation	
Oscillator	$\hat{x}$	$\left|\hat{F}\right|/{\left|\hat{F}\right|}_{\max}$	$\psi_{\hat{F}}{/}^{\circ }$
1	1	0.061	154.8
2	0	0.058	−178.7
3	0	0.052	−178.7
4	0	0.058	−178.7
5	0	1	−178.7

**Table 4:** Complex excitation and displacement amplitudes for adapted approach to complete vibration localization in the active area: Computation of a reduced excitation vector $\hat{F}$ for complete vibration localization within the active elements; the displacement of the base is not of interest: ${\hat{x}}_{\mathrm{target}}^{\mathrm{red}}={\left[\begin{array}{cccc} 1 & 0 & 0 & 0 \end{array}\right]}^{T}$. By taking the reduced excitation vector and setting excitation at the base to zero, the displacements for the entire system can be calculated. We can observe complete vibration localization within the active elements 1–4. In turn, displacement of oscillator 5 can be seen. Calculations are performed for ε=0.058 and $\omega \;=\;\omega^{\mathrm{res},\mathrm{exc}}$.

		Reduced Required Excitation			Resulting Displacement	
Oscillator	${\hat{x}}^{\mathrm{red}}$	$\left|{\hat{F}}^{\mathrm{red}}\right|/{\left|{\hat{F}}^{\mathrm{red}}\right|}_{\max}$	$\psi_{\hat{F}}{/}^{\circ }$	$\hat{F}$	$\left|\hat{x}\right|/{\left|\hat{x}\right|}_{\max}$	$\psi_{\hat{x}}{/}^{\circ }$
1	1	1	92.5	${\hat{F}}_{1}^{\mathrm{red}}$	1	0
2	0	0.259	120.2	${\hat{F}}_{2}^{\mathrm{red}}$	0	-
3	0	0.231	81.2	${\hat{F}}_{3}^{\mathrm{red}}$	0	-
4	0	0.259	120.2	${\hat{F}}_{4}^{\mathrm{red}}$	0	-
5	-	-	-	0	0.054	−171.6

**Table 5:** Exchange of excitation: Complex displacement amplitudes for $\omega \;=\;\omega^{\mathrm{res},\mathrm{exc}}\;=\;2\pi f^{\mathrm{res},\mathrm{exc}}$ for the case of close frequencies in the transition zone (ε=0.056). Values are shown for excitation at various oscillators. The corresponding resonance frequency of the excited oscillator is given. Note the deviation as excitation is interchanged between oscillators. Refer to Appendix E for the corresponding operational deflection shapes.

	Excitation at Oscillator:							
	1		2		3		4	
Oscillator	$\left|\hat{x}\right|/{\left|\hat{x}\right|}_{\max}$	$\psi_{\hat{x}}{/}^{\circ }$	$\left|\hat{x}\right|/{\left|\hat{x}\right|}_{\max}$	$\psi_{\hat{x}}{/}^{\circ }$	$\left|\hat{x}\right|/{\left|\hat{x}\right|}_{\max}$	$\psi_{\hat{x}}{/}^{\circ }$	$\left|\hat{x}\right|/{\left|\hat{x}\right|}_{\max}$	$\psi_{\hat{x}}{/}^{\circ }$
1 *	1	−90.3	0.042	164.0	0.084	105.0	0.042	164.0
2	0.115	94.1	1	−90.1	0.177	102.7	0.239	56.1
3	0.056	75.6	0.177	111.7	1	−90.6	0.177	111.7
4	0.115	94.1	0.239	56.1	0.177	102.7	1	−90.1
5	0.03	95.3	0.035	100.5	0.032	87.5	0.035	100.5
$f^{\mathrm{res},\mathrm{exc}}$	54.96		52.93		53.02		52.93	

* Disordered oscillator.

**Figure A1:**
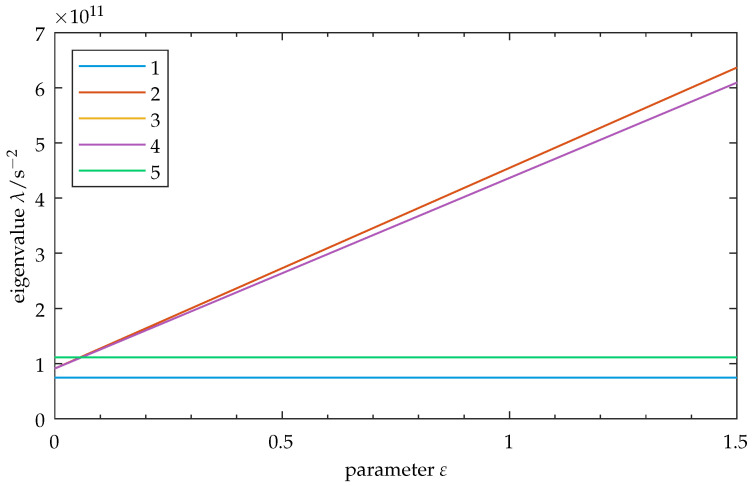
Symmetric model: Eigenvalues λ for modes 1–5 as a function of the parameter ε.

**Figure A2:**
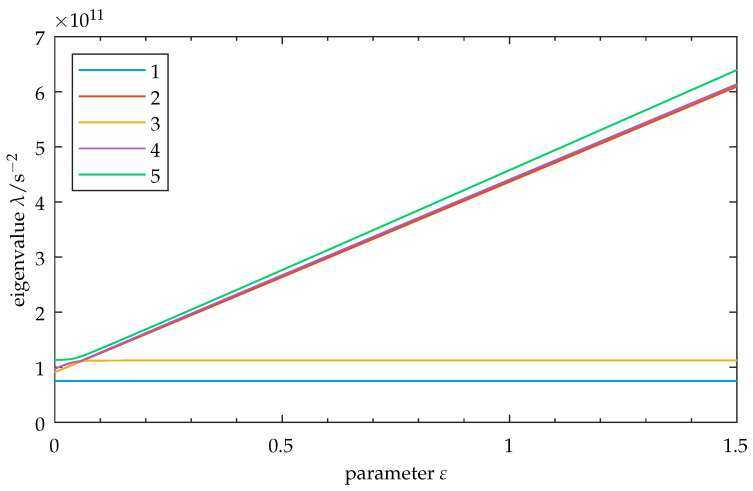
Disordered model: Eigenvalues λ for modes 1–5 as a function of the parameter ε. Disorder is introduced into the initial state (cf. Equation (20)).

**Figure A3:**
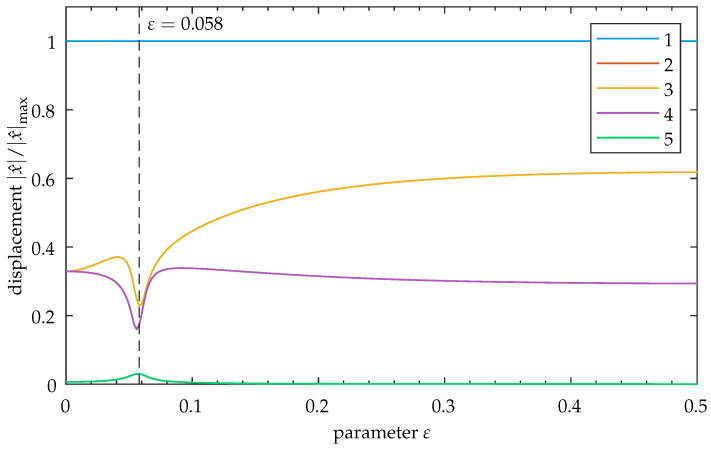
Symmetric model, excitation at oscillator 1: Relative displacement amplitudes $\left|\hat{x}\right|/{\left|\hat{x}\right|}_{\max}$ for oscillators 1–5 as a function of the parameter ε. The vertical dashed line marks the point of eigenvalue crossing at ε=0.058, as discussed in Section 3.2.3. Observe the inverse relation between strength of coupling (represented by the parameter ε) and vibration localization: As ε increases, the relative displacement amplitudes of the non-excited elements increase, reducing vibration localization. Note that the upper limit of ε is set to 0.5 for clarity.

**Figure A4:**
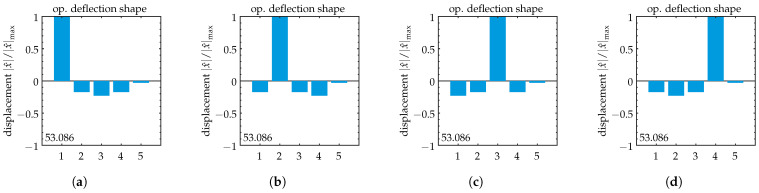
Symmetric model: Simplified operational deflection shapes for ε=0.058. Operating frequencies are given in [kHz] within each subplot. Forced vibration localization is displayed for various excitation cases. (**a**) Excitation at oscillator 1, (**b**) excitation at oscillator 2, (**c**) excitation at oscillator 3, (**d**) excitation at oscillator 4. Observe the *imprint* of the relative vibration displacements. Its “orientation” toward the excited oscillator is retained as different oscillators are excited. This results in varying displacement amplitudes *at an observed oscillator*.

**Figure A5:**
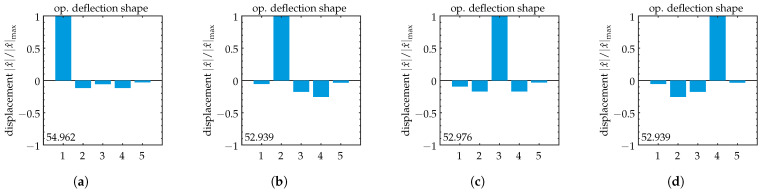
Disordered model: Simplified operational deflection shapes for ε=0.056. Disorder is introduced into the initial state at oscillator 1 (cf. Equation (20)). Operating frequencies are given in [kHz] within each subplot. Forced vibration localization is displayed for various excitation cases. (**a**) Excitation at oscillator 1, (**b**) excitation at oscillator 2, (**c**) excitation at oscillator 3, (**d**) excitation at oscillator 4. Observe the difference in relative displacement amplitudes for the different excitation cases. This is accompanied by a change in resonance frequency.

## Data Availability

The original contributions presented in this study are included in the article. Further inquiries can be directed to the corresponding author.

## Abbreviations

The following abbreviations are used in this manuscript:

DOF	degree of freedom
FRF	frequency-response function
MEMS	micro-electromechanical system

## Appendix A. Matrices

The equations of motion for free vibration of the 5-DOF system described in Section 2.4 are

(A1) $$\begin{array}{c} \begin{array}{cc} \; & m_{1}\;{\ddot{x}}_{1}+\left(k_{1}+k_{12}+k_{13}+k_{41}\right)x_{1}-k_{12}\;x_{2}-k_{13}\;x_{3}\;-k_{41}\;x_{4}-k_{1}\;x_{5}=0,\\ \; & m_{2}\;{\ddot{x}}_{2}-k_{12}\;x_{1}+\left(k_{2}+k_{23}+k_{24}+k_{12}\right)\;x_{2}-k_{23}\;x_{3}-k_{24}\;x_{4}-k_{2}\;x_{5}=0,\\ \; & m_{3}\;{\ddot{x}}_{3}-k_{13}\;x_{1}-k_{23}\;x_{2}+\left(k_{3}+k_{34}+k_{13}+k_{23}\right)\;x_{3}-k_{34}\;x_{4}-k_{3}\;x_{5}=0,\\ \; & m_{4}\;{\ddot{x}}_{4}-k_{41}\;x_{1}-k_{24}\;x_{2}-k_{34}\;x_{3}+\left(k_{4}+k_{41}+k_{24}+k_{34}\right)\;x_{4}-k_{4}\;x_{5}=0,\\ \; & m_{5}\;{\ddot{x}}_{5}-k_{1}\;x_{1}-k_{2}\;x_{2}-k_{3}\;x_{3}-k_{4}\;x_{4}+\left(k_{1}+k_{2}+k_{3}+k_{4}+k_{5}\right)\;x_{5}=0. \end{array} \end{array}$$

Putting Equation (A1) into matrix notation yields the system matrices:

(A2) $$\begin{array}{cc} M & =\left[\begin{array}{ccccc} m_{1} & 0 & 0 & 0 & 0\\ 0 & m_{2} & 0 & 0 & 0\\ 0 & 0 & m_{3} & 0 & 0\\ 0 & 0 & 0 & m_{4} & 0\\ 0 & 0 & 0 & 0 & m_{5} \end{array}\right] \end{array}$$

(A3) $$\begin{array}{cc} K & =\left[\begin{array}{ccccc} k_{1}+k_{12}+k_{13}+k_{41} & -k_{12} & -k_{13} & -k_{41} & -k_{1}\\ -k_{12} & k_{2}+k_{23}+k_{24}+k_{12} & -k_{23} & -k_{24} & -k_{2}\\ -k_{13} & -k_{23} & k_{3}+k_{34}+k_{13}+k_{23} & -k_{34} & -k_{3}\\ -k_{41} & -k_{24} & -k_{34} & k_{4}+k_{41}+k_{24}+k_{34} & -k_{4}\\ -k_{1} & -k_{2} & -k_{3} & -k_{4} & k_{1}+k_{2}+k_{3}+k_{4}+k_{5} \end{array}\right] \end{array}$$

(A4) $$\begin{array}{cc} x & =\left[\begin{array}{c} x_{1}\\ x_{2}\\ x_{3}\\ x_{4}\\ x_{5} \end{array}\right] \end{array}$$

The introduction of viscous damping into the 5-DOF system gives a damping matrix

(A5) $$\begin{array}{cc} C & =\beta \left[\begin{array}{ccccc} k_{1}+k_{12}+k_{13}+k_{41} & -k_{12} & -k_{13} & -k_{41} & -k_{1}\\ -k_{12} & k_{2}+k_{23}+k_{24}+k_{12} & -k_{23} & -k_{24} & -k_{2}\\ -k_{13} & -k_{23} & k_{3}+k_{34}+k_{13}+k_{23} & -k_{34} & -k_{3}\\ -k_{41} & -k_{24} & -k_{34} & k_{4}+k_{41}+k_{24}+k_{34} & -k_{4}\\ -k_{1} & -k_{2} & -k_{3} & -k_{4} & k_{1}+k_{2}+k_{3}+k_{4}+k_{5} \end{array}\right], \end{array}$$

where β is defined in Table 1.

## Appendix B. Parameter Variation: Full Range Plots

Range for parameter ε:[0,1.5].
